# Fructose and NAFLD: The Multifaceted Aspects of Fructose Metabolism

**DOI:** 10.3390/nu9030230

**Published:** 2017-03-03

**Authors:** Prasanthi Jegatheesan, Jean-Pascal De Bandt

**Affiliations:** 1 Department of Physiology, University of Lausanne, CH-1005 Lausanne, Switzerland; 2 EA4466, Faculty of Pharmacy, Paris Descartes University, Sorbonne Paris Cité, 75006 Paris, France; jean-pascal.de-bandt@univ-paris5.fr; 3 Clinical Chemistry Department, Hôpitaux Universitaires Paris Centre, Assistance Publique-Hôpitaux de Paris, 75679 Paris, France

**Keywords:** fructose, nonalcoholic fatty liver disease, liver, gut, muscle

## Abstract

Among various factors, such as an unhealthy diet or a sedentarity lifestyle, excessive fructose consumption is known to favor nonalcoholic fatty liver disease (NAFLD), as fructose is both a substrate and an inducer of hepatic de novo lipogenesis. The present review presents some well-established mechanisms and new clues to better understand the pathophysiology of fructose-induced NAFLD. Beyond its lipogenic effect, fructose intake is also at the onset of hepatic inflammation and cellular stress, such as oxidative and endoplasmic stress, that are key factors contributing to the progression of simple steatosis to nonalcoholic steatohepatitis (NASH). Beyond its hepatic effects, this carbohydrate may exert direct and indirect effects at the peripheral level. Excessive fructose consumption is associated, for example, with the release by the liver of several key mediators leading to alterations in the communication between the liver and the gut, muscles, and adipose tissue and to disease aggravation. These multifaceted aspects of fructose properties are in part specific to fructose, but are also shared in part with sucrose and glucose present in energy–dense beverages and foods. All these aspects must be taken into account in the development of new therapeutic strategies and thereby to better prevent NAFLD.

## 1. Introduction

Nonalcoholic-fatty liver disease (NAFLD) represents a spectrum of disorders ranging from simple steatosis to nonalcoholic steatohepatitis (NASH), which can progress to fibrosis, cirrhosis, and liver cancer [1]. Its prevalence increases with that of type 2 diabetes, obesity, and metabolic syndrome [2] and is considered to be on average 20%–25%. Although several factors may contribute to NAFLD [3], fructose consumption is considered as a key player in the development of this disease [4,5], and it has repeatedly been reported to induce NAFLD in humans [6,7] and rodents [8,9]. A significant consumption of fructose leads to hepatic lipid accumulation and steatosis, steatosis being considered pathological when an abnormal accumulation of lipid droplets is observed in the cytoplasm of at least 5% of hepatocytes [4]. At this point, hepatic steatosis (HS) may be reversed through nutritional and physical exercise approaches [10,11]. Adversely, chronic consumption of fructose promotes several processes such as inflammation and cellular stress, which is responsible for the irreversibility of hepatic disorders and the progression of the disease [4]. The current review also provides new insights into the metabolic consequences of high fructose intake on peripheral tissues contributing to NAFLD progression.

## 2. Fructose and Hepatic Steatosis

An activation of the lipogenic program already occurs after a single load of fructose, leading to hepatic lipid accumulation [12,13,14]. As described below, this is the consequence of facilitated hepatic fructose metabolism for lipid synthesis and of the activation of signaling pathways whereby fructose promotes de novo lipogenesis (DNL).

### 2.1. Fructose as a Substrate of Hepatic de novo Lipogenesis

Fructose is subjected to rapid unregulated entry into the liver mainly via the glucose transporter 2 (GLUT2). At cell level, this carbohydrate is preferentially converted into fructose-1-phosphate (F1P) by fructokinase, which presents a high affinity for fructose, is not controlled by insulin, and is induced by fructose [15]. Thereafter, phosphotrioses produced from F1P through the action of aldolase B can be converted into glucose, lactate, and fatty acids [16]. While the lipogenic pathway is quantitatively minor in physiological situations, it becomes very active after an acute fructose load [12,17] as the flux of fructose carbons into lipogenic precursors increases, since the formation of F1P bypass the glycolysis regulatory site of phosphofructokinase1. Unregulated entry and metabolism of fructose into hepatocytes explain why, with high fructose diets, significant amounts of this carbohydrate continue to enter glycolysis and lead to excess acetyl-CoA production, relative to liver oxidative capacities, thus promoting DNL. High consumption of fructose also leads, by saturating the glycolytic pathway, to an accumulation of glycolysis intermediates which can be converted to glycerol-3-phosphate used in triglyceride (TG) synthesis.

### 2.2. Fructose as an Inducer of De Novo Lipogenesis

Chronic intake of fructose increases DNL by activating several key transcription factors [12] such as Sterol Response Element Binding Protein 1c (SREBP1c) and Carbohydrate-Responsive Element-Binding Protein (ChREBP) [17,18]. As a consequence, their key target enzymes regulating lipid synthesis, such as Fatty Acid Synthase (FASN) and Acetyl-CoA Carboxylase (ACC), also increase as shown for example in rodents submitted to a 60% high fructose diet for eight weeks [18] or to a western diet where fructose is provided as a 30%-fructose containing beverage for eight weeks [19]. 

Thus, as fructose is both substrate and activator of DNL, it appears as the most potent lipogenic carbohydrate contributing to the development of liver steatosis.

## 3. Fructose and Disease Progression

Fructose by itself or via increased DNL may promote oxidative stress, in part via mitochondrial dysfunction and endoplasmic reticulum (ER) stress, both contributing to the development of an inflammatory process and the progression of simple steatosis to NASH.

### 3.1. Fructose and Oxidative Stress

Fructose induces oxidative stress *via* several mechanisms. First, because fructose is structurally different from glucose, it can promote more hepatocellular damage. Acute fructose load induces protein fructosylation. This reaction is non-enzymatic and is seven times faster than glycation by glucose. In addition, fructose generates 100 times more reactive oxygen species (ROS) than glucose [4]. Compared with glucose, prolonged fructose feeding in mice led to a higher hepatic accumulation of carboxymethylysine, a glycation product that, for example, can interact with SREBP-cleavage activating protein to induce sustained SREBP1c activation [20].

Second, fructose phosphorylation in the liver consumes adenosine triphosphate (ATP): As phosphorylation by fructokinase is fast and the cleavage reaction by aldolase B relatively slow, an excess of fructose could cause hepatic phosphate deficiency, leading to AMP accumulation with resulting increased uric acid synthesis [7,21]. Uric acid in turn stimulates the production of ROS [22] via the activation of Transforming Growth Factor β and nicotinamide adenine dinucleotide phosphate (NADPH) oxidase 4 ([23].

Third, hepatic metabolism of fructose generates other molecules such as methylglyoxal (MG), a potent glycating agent leading to cellular stress and altered insulin signaling [24]. In condition of standard feeding, MG formation rate represents 0.1%–0.4% of glycolytic flux [25] but accelerated glycolytic flux with fructose increases MG formation.

Last, mitochondrial dysfunction may also be induced by the lipotoxicity related to the fructose-induced perturbation of hepatic lipid metabolism [8]. Mechanisms involved may be (i) a decrease in lipid degradation due to a lower expression of Peroxisome Proliferator-Activated Receptor α (PPARα) that regulates genes involved in β-oxidation such as Carnitine Palmitoyl Transferase 1 (CPT1) [18], a lower expression of the peroxisomal proliferator-activated receptor-gamma coactivator-1alpha (PGC-1α) (a mitochondrial-biogenic protein) [26]; and (ii) a decrease in lipid clearance due to a lower expression of Microsomal Triglyceride Transfer Protein (MTP) [18] involved in Very Low Density Lipoprotein (VLDL) production. However, the exact mechanisms remain debated as, in some studies, an enhancement of beta-oxidation and VLDL-clearance after fructose consumption has been reported, suggesting that hepatic lipid accumulation mainly results from uncontrolled DNL [27]. As a result, the disequilibrium between DNL and VLDL release may promote alterations of the respiratory chain and to the uncoupling of oxidative phosphorylation with excess ROS production [28,29]. Mitochondria and ER being associated through mitochondria-associated ER-membrane plays a key role in calcium signaling and lipids transfer, ROS overproduction by mitochondria contributes to ER stress, and hepatic inflammation, two processes addressed in the following sections.

### 3.2. Fructose and Endoplasmic Reticulum Stress

Studies pointed to ER stress as a mechanism favoring HS progression to NASH [30]. Chronic fructose consumption leads to a higher solicitation of the ER via the stimulation of lipid metabolism and of VLDL-TG production. ER membrane proteins may be fructosylated, or lipids may accumulate into ER membrane leading to ER stress and the unfolded protein response (UPR). Although UPR activation first allows the restoration of ER homeostasis, during sustained fructose exposure ER stress becomes chronic leading, to inflammation, oxidative stress, and apoptosis [31,32]. This also contributes to the progression of hepatic steatosis and of insulin resistance [33]: ER stress further interferes with lipid metabolism in the liver by activating DNL, via the protein kinase activated by dsRNA (PKR)-related Endoplasmic Reticulum Kinase (PERK)/eukaryotic translation Initiation Factor 2α (eIF2α)/Activating Transcription Factor 4 (ATF4) pathway and by limiting the formation and secretion of VLDL, via Inositol Requiring Enzyme 1 (IRE1) pathway. ER stress also acts indirectly on the accumulation of TG in the liver by inducing hepatic and adipose tissue insulin resistance. Furthermore, ER stress promotes the activation of transcription factors Janus kinase (JNK), Nuclear Factor κB (NFκB), ChREBP, SREBP, and CCAAT/enhancer-binding protein homologous protein (CHOP), which are involved in inflammatory processes and cell death and play an important role in the progression of NAFLD [33].

### 3.3. Fructose and Inflammation

The contribution of fructose diet to the inflammatory process is well established [34]. The specific role of hepatic fructose metabolism in liver inflammation is suggested by the protective effect of fructokinase knockout against high-fat high-sucrose-induced steatohepatitis [3]. Ectopic liver fat accumulation increases hepatocytes vulnerability to cellular stress, therefore initiating an inflammatory process [35]. In parallel, cellular stress can be exacerbated by toll-like receptor 4 (TLR4) activation-induced inflammation in Kupffer cells since fructose has been shown to promote the synthesis of saturated fatty acids such as palmitate, which are able to activate TLR4 receptors in the liver [36]. The activation of the TLR4/inducible nitric oxide synthase (iNOS)/NFκB pathway induces oxidative stress in hepatocytes via the production of pro-inflammatory cytokines, such as tumor necrosis factor (TNF) α by Kupffer cells. These phenomena are reinforced by the lipid-induced increase in the proportion of “conventional” pro-inflammatory M1 macrophages relative to “alternate” anti-inflammatory M2 macrophages [37]. In a study in 427 patients with NAFLD, fructose consumption has been shown to be associated with increased hepatic fibrosis, in keeping with a fructose-induced increase in hepatic inflammation and ER stress [7]. Last, fructose also modulates liver inflammation by inducing dysbiosis as discussed in detail below.

The superimposition of ER stress and inflammation may lead to the production of various mediators such as cytokines, hepatokines, carbohydrates, and lipid derivatives collectively known as DAMPs (damage associated molecular pattern) that signal at the whole-body level and contribute to alterations in whole body metabolism. Many studies, both in animal models [18,19] and more recently in patients with NASH, show that abnormal hepatokines production also plays a key role in the pathogenesis of NASH [38,39]. Hepatokines such as Fetuin A, Fibroblast growth factor 21 (FGF-21), Leucocyte cell-derived chemotaxin 2 (LECT2), and Angiopoietin-like protein (ANGPTL) released by the steatotic liver may contribute to peripheral organ dysfunction [40,41].

## 4. Fructose and Interorgan Cross-Talks

**Figure 1:**
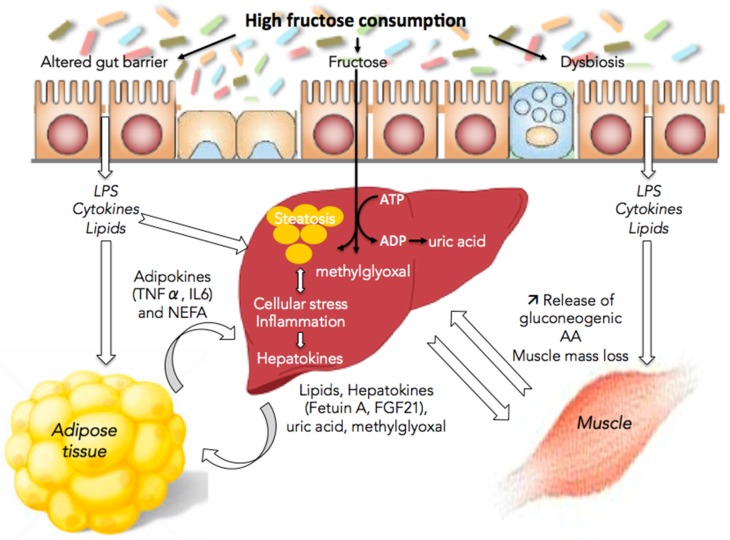
Fructose and nonalcoholic fatty liver disease (NAFLD): the multifaceted aspects of fructose metabolism. Excessive fructose consumption is associated with hepatic steatosis, cellular stress and inflammation. This is responsible for the release by the liver of lipids, methyglyoxal, uric acid, and hepatokines leading to alterations in the communication between the liver and the gut, muscles, and adipose tissue and to disease aggravation. LPS, lipopolysaccharides; TNFα, tumor necrosis factor α; IL6, interleukin-6; NEFA, non-esterified fatty acid; ATP, adenosine triphosphate; ADP, adenosine diphosphate; FGF21, fibroblast growth factor 21; AA, amino acids.

The following sections review some of the mechanisms whereby fructose directly or indirectly, through the release of lipids, hepatokines, and uric acid into the blood, leads to alterations in gut, muscle, and adipose tissue functions (Figure 1). 

### 4.1. Fructose and the Gut/Liver Axis

The progression of HS to NASH is also influenced by gut function and the possible translocation of bacterial compounds due to a compromised intestinal barrier [42,43]. First, insulin resistance by itself is already associated with alterations in gut permeability [44]. In parallel, patients with NAFLD present a dysbiosis characterized by an increase in *Clostridium coccoides* and a decrease in Bacteroides/Prevotella [45]. Fructose-induced NAFLD is also associated with changes in microbiota composition [46] that alters gut permeability by reducing expression of tight junction proteins [19,47]. As a consequence of this alteration in gut barrier function and of the dysbiosis, NASH, and cirrhotic patients present an increase in endotoxin translocation [45,48]. The ensuing activation of TLR4 in Kupffer cells and infiltrated monocytes worsen innate and adaptive immune responses. Liver exposition to endotoxins such as lipopolysaccharides (LPS) may induce a chronic inflammation associated with a recruitment of neutrophils that release ROS, proteases, lipocalin-2, and enzymes leading to an aggravation of liver injuries [8,49]. LPS and oxidative stress also activate stellar cells leading to fibrosis. Thereafter, cytokines activate several signaling pathways such as the pro-apoptotic pathways [50]. Gut barrier alteration also promotes hepatic macrophages polarization to M1 phenotype further favoring inflammatory liver injury [51]. 

Last, enterocytes also metabolize a small part of fructose into lactate, glucose, and also TG. Theytaz et al. [21] demonstrate an increase in both ^13^C-palmitate chylomicron and ^13^C-palmitate VLDL-TG concentrations after a ^13^C-fructose load in non-obese, young human subjects. This may contribute to the alteration of metabolism and ultimately of liver function [21,52]. 

Thus, it seems important to consider the gut-liver axis in the management of NASH or of other NAFLD stages.

### 4.2. Fructose and Adipose Tissue/Liver Axis

Visceral fat mass increases with fructose diet in humans [53] as well as in experimental models [18,54]. This suggests either a direct fructose metabolism in visceral adipocytes, which may be exposed to higher fructose concentrations than subcutaneous adipocytes due to anastomosis between portal hepatic and systemic splanchnic circulation, or an indirect effect through an accumulation of lipid originating from the liver. Vràna et al. [55] showed an inhibition of DNL in adipose tissue in fructose-fed rats. The increase in fructose-derived MG production by the liver may play a role as Masterjohn et al. [56] showed that fructose-fed rats display an accumulation of MG in epididymal adipose tissue. MG alters insulin signaling pathway in visceral adipose tissue in vivo [57]. In vitro, fructose increases adipogenesis and, conversely, the inhibition of fructose transport in mice is associated with reduced epididymal adipose tissue [58]. Together these data underline fructose influence on visceral adipose tissue but data in human are missing.

Owing to this adipogenic effects, adipokines and cytokines profile would also be changed by fructose diet. The consequences of this increased visceral adiposity are elevated circulating free fatty acids and proinflammatory mediators. Due to the anatomic proximity and the portal circulation, this will clearly alter liver function but also other the function of peripheral organs leading to an aggravation of the metabolic disorders [34].

### 4.3. Fructose and Muscle/Liver Axis

A high-fructose diet is associated with modifications in muscle function [59] in humans [26] and in rodents [60]. Mechanisms involved in diet-induced sarcopenia may be (i) a decrease in mechanistic target of rapamycine complex (mTORC) 1 activity, and thereafter in protein synthesis [61]; and (ii) inflammation [62]. Recent studies in fructose-fed rats have shown an association between NAFLD and sarcopenia [63]. This is a key factor involved in disease progression to NASH as the muscle heavily contributes to energy homeostasis [64]. Gatineau et al. [65] recently showed in aged rats that sucrose-fed animals lost significantly more lean body mass and retained more fat mass than starch-fed rats and presented lower meal-induced stimulation of muscle protein synthesis.

Disorders of nitrogen homeostasis in situations of stimulated DNL may be an early event following excessive fructose consumption [12,51] as excess fructose may alter liver-muscle axis via its metabolism or via DNL-associated RE stress leading to increased production by the liver of catabolic effectors. First, the increase in lipid flux observed with fructose-enriched diet contributes to alter muscle insulin sensitivity [59,66,67]. Second, as previously described, excess fructose may lead to a saturation of its normal metabolism with adverse consequences in terms of increased hepatic release of MG or uric acid. In vitro studies show that MG inhibits insulin signaling in muscle [68]. High fructose diet under hypercaloric feeding conditions has been shown to induce hyperuricemia that contributes to metabolic disorders [69,70]. Uric acid inhibits muscle insulin signaling and induces insulin resistance in mice [71] as well as in severely obese subjects [72]. Third, hepatic ER is associated with enhanced production of pro-inflammatory cytokines and hepatokines suspected to be involved in alterations in energy homeostasis and insulin-resistance. ER stress markedly stimulates liver production of Fetuin A [73] and of insulin-like growth factor binding protein 1 (IGFBP1) [74]. Fetuin A is an endogenous inhibitor of the insulin receptor tyrosine kinase in muscle [75], while IGFBP1 is a modulator of insulin-like growth factor 1 (IGF-1) action associated with hyperinsulinemia and glucose intolerance [76]. ER stress modulates fibroblast growth factor 21 (FGF21) expression in the liver [77]. FGF21 is a mediator mainly produced by the liver that contributes to the regulation of peripheral energy metabolism and insulin sensitivity [78]. It is now recognized as a key player in the adaptive response to starvation and feeding [79]. Last, fructose consumption leads to decreased liver production of anabolic factors such as insulin-like growth factor (IGF)1 [64]. 

Another factor contributing to these alterations of protein metabolism is a reorientation of AA fluxes as suggested by NAFLD-associated changes in plasma amino acids (AAs) profile [18]. In hypertriglyceridemic patients, fructose increased plasma arterial AA concentrations but also their splanchnic extraction [80]. These interorgan AA fluxes probably correspond to a reorientation of AAs towards the liver in order to enable the synthesis of inflammatory proteins and the elevated gluconeogenesis. In situations of fructose overfeeding, energy metabolism would be oriented towards an increase in gluconeogenesis and DNL and a decrease in lipid catabolism. Conversely, a regulatory role of AA availability on liver DNL has been shown in experimental and human studies as increased AA availability prevents hepatic lipid accumulation via (i) a decrease in DNL through decreased gene expression of ChREBP, SREBP-1c and Fas (ii) an increase in ß-oxidation through increased gene expression of PPARα; and (iii) an enhance in VLDL production through increased gene expression of MTP [18]. AA supplementation has also been shown to decrease gene expression of TLR4 and interleukin-6 (IL6) in liver and to prevent the loss in lean body mass in fructose-fed rats [18]. The basis for this interaction between DNL, AA availability, and protein homeostasis needs to be confirmed in humans. Interestingly, in healthy volunteers, essential AA supplementation decreased fructose-induced intrahepatic lipid accumulation [21].

## 5. Specific or Indirect Effect of Fructose

The above-mentioned peripheral manifestations associated with fructose feeding and several short-term studies, using ^13^C-fructose as metabolic tracer, suggest a specific effect of fructose. Although it has long been taught that fructose is mainly metabolized in the liver [16], a small part of this carbohydrate may bypass liver extraction and be metabolized in extrahepatic cells since various cells, including neurons, express fructose transporter GLUT5 and enzymes involved in its metabolism [20,56,81]. However, data in human are missing.

Moreover, the exact contribution of fructose intake is frequently blurred by the associated imbalance in energy homeostasis. Fructose is often consumed in diets also rich in glucose and lipids. Although this carbohydrate is more harmful than glucose as it is more lipogenic and its metabolism differs from that of glucose [8,45], its effects are amplified when it is associated with glucose [82]. Indeed, fructose effects are more severe when consumed in the form of disaccharides (i.e., sucrose composed of equal parts of fructose and glucose) or associated with other macromolecules such as lipids [19,83].

## 6. Fructose and NAFLD Management

Lifestyle changes, including physical activity and balanced diets, are the initial treatment of steatosis, especially when they enable to lose weight. In NASH patients, a 3% to 7% weight loss is associated with decreased hepatic steatosis [84]:

Exercise prevents fructose-induced hypertriglyceridemia in healthy subjects and promotes a decrease in hepatic TG content [85]. In patients with NASH, markers of disease severity are decreased after 200 min per week of moderate intensity physical activity for 48 weeks, associated with a balanced diet [86].

Apart from limiting caloric intake, these patients should avoid a diet rich in saturated fatty acids, sucrose, and alcohol [3,87]. For example, the Mediterranean diet rich in mono-unsaturated fatty acids may be effective [88]. It has been shown to reduce liver steatosis and improve insulin sensitivity in patients with NAFLD without diabetes [89]. Diets enriched with omega-3 polyunsaturated fatty acids may also reduce steatosis [90]. 

A more dramatic strategy to induce weight loss is bariatric surgery. It is an effective procedure to improve insulin resistance and glucose metabolism primarily by reducing calorie intake, thereby reducing body weight and liver steatosis [91].

Other possible alternatives are pro- and prebiotics, which are of growing interest in the management of these patients because of their effect on gut microbiota and/or gut barrier function. For example, *Lactobacillus rhamnosus* GG protects against the development of fructose-induced NAFLD via the preservation of gut microbiota thus restoring the intestinal barrier via increased expression of Claudine-1 and Occludine tight junction proteins [47]. Last, there is an increasing interest in natural products and plant extracts that could be effective on some aspects on fructose-induced NAFLD [92,93]. However, their clinical effectiveness remain to be evaluated.

In NAFLD patients with metabolic syndrome or type 2 diabetes, lipid-lowering therapy or insulin-sensitizing agents have been proposed. Statins, fenofibrate, and ezetimibe treatments result in only modest improvement to liver damage in NASH patients. The effects of the insulin-sensitizer metformin are debated: while Bergheim showed that metformin protects mice against fructose-induced NAFLD [94], metformin does not improve the histological alterations observed in people with NASH [95]. Concerning thiazolidinediones, their side effects preclude their use in NAFLD [95]. 

## 7. Conclusions

Based on its specific splanchnic (predominantly hepatic) metabolism, on its lipogenic potential, and on its high consumption in modern diets, fructose appears as one major factor not only of the initiation of hepatic steatosis, but also of its progression to NASH and more severe stages of the disease. Understanding its metabolism may provide novel opportunities for therapeutic intervention.

## Abbreviations

AA: amino acid; ADP, Adenosine Diphosphate; ACC: Acetyl CoA Carboxylase; ANGPTL: Angiopoietin-like protein; ATF4: Activating Transcription Factor 4; ATP: Adenosine Triphosphate; CHOP: CCAAT-enhancer-binding protein homologous protein; ChREBP: Carbohydrate-Responsive Element-Binding Protein; CPT1: Carnitine Palmitoyl Transferase 1; DAMP: damage associated molecular pattern; DNL: de novo lipogenesis; eIF2α: eukaryotic translation Initiation Factor 2α; ER: endoplasmic reticulum; F1P: fructose-1-phosphate; FASN: Fatty Acid Synthase; FGF-21: Fibroblast Growth Factor 21; GLUT: glucose transporter; HS : hepatic steatosis; IGF: insulin-like growth factor; IGFBP: IGF binding protein1; iNOS: inducible nitric oxide synthase; IL6: Interleukin-6; IRE1: Inositol Requiring Enzyme 1; JNK: Janus kinase; LECT2: Leucocyte cell-derived chemotaxin 2; MG: methylglyoxal; mTORC: mechanistic target of rapamycine complex complex; MTP: microsomal Triglyceride transfer Protein; NADPH: Nicotinamide Adenine Dinucleotide Phosphate; NAFLD: nonalcoholic fatty liver disease; NASH: nonalcoholic steatohepatitis; NEFA, Non-Esterified Fatty Acid; NFκB: Nuclear Factor κB; PERK: PKR-related Endoplasmic Reticulum Kinase; PKR: Protein Kinase Activated by dsRNA; PPARα: Peroxisome Proliferator-Activated Receptor α; ROS: reactive oxygen species; SREBP1c: Sterol Response Element Binding Protein 1c; TG: triglycerides; TLR4: toll-like receptor 4; TNF: tumor necrosis factor; UPR: unfolded protein response; VLDL: Very Low Density Lipoprotein.
